# Integrated community case management of malaria and pneumonia increases prompt and appropriate treatment for pneumonia symptoms in children under five years in Eastern Uganda

**DOI:** 10.1186/1475-2875-12-340

**Published:** 2013-09-22

**Authors:** Joan N Kalyango, Tobias Alfven, Stefan Peterson, Kevin Mugenyi, Charles Karamagi, Elizeus Rutebemberwa

**Affiliations:** 1 Department of Public Health Sciences, Global Health (IHCAR), Karolinska Institutet, SE 17177, Stockholm, Sweden; 2 Clinical Epidemiology Unit, Makerere University College of Health Sciences, P.O. Box 7072, Kampala, Uganda; 3 Department of Pharmacy, Makerere University College of Health Sciences, P.O. Box 7072, Kampala, Uganda; 4 Department of Paediatrics, Sach’s Children’s Hospital, Södersjukhuset, Stockholm, Sweden; 5 International Maternal and Child Health, Department of Women and Children’s Health, Uppsala University, Uppsala, Sweden; 6 African Field Epidemiology Network (AFENET), P.O Box 12874, Kampala, Uganda; 7 Department of Paediatrics and Child Health, Makerere University College of Health Sciences, P.O. Box 7072, Kampala, Uganda; 8 Department of Health Policy, Planning and Management, School of Public Health, Makerere University College of Health Sciences, P.O. Box 7072, Kampala, Uganda

**Keywords:** CHW, ICCM, Health System Research, Prompt treatment, Appropriate treatment, Treatment outcomes, Malaria, Pneumonia, Children, CMDs

## Abstract

**Background:**

Efforts to improve access to treatment for common illnesses in children less than five years initially targeted malaria alone under the home management of malaria strategy. However under this strategy, children with other illnesses were often wrongly treated with anti-malarials. Integrated community case management of common childhood illnesses is now recommended but its effect on promptness of appropriate pneumonia treatment is unclear.

**Objectives:**

To determine the effect of integrated malaria and pneumonia management on receiving prompt and appropriate antibiotics for pneumonia symptoms and treatment outcomes as well as determine associated factors.

**Methods:**

A follow-up study was nested within a cluster-randomized trial that compared under-five mortality in areas where community health workers (CHWs) treated children with malaria and pneumonia (intervention areas) and where they treated children with malaria only (control areas). Children treated by CHWs were enrolled on the day of seeking treatment from CHWs (609 intervention, 667 control) and demographic, illness, and treatment seeking information was collected. Further information on illness and treatment outcomes was collected on day four. The primary outcome was prompt and appropriate antibiotics for pneumonia symptoms and the secondary outcome was treatment outcomes on day four.

**Results:**

Children in the intervention areas were more likely to receive prompt and appropriate antibiotics for pneumonia symptoms compared to children in the control areas (RR = 3.51, 95%CI = 1.75-7.03). Children in the intervention areas were also less likely to have temperature ≥37.5°C on day four (RR = 0.29, 95%CI = 0.11-0.78). The decrease in fast breathing between day one and four was greater in the intervention (9.2%) compared to the control areas (4.2%, p-value = 0.01).

**Conclusions:**

Integrated community management of malaria and pneumonia increases prompt and appropriate treatment for pneumonia symptoms and improves treatment outcomes.

**Trial registration:**

ISRCTN: ISRCTN52966230

## Background

Although global mortality in children less than five years reduced from 12 million deaths in 1990 to 6.9 million deaths in 2011 [1], the number of deaths is still high and many countries are at risk of not achieving Millennium Development Goal four [2]. This is more so in Africa and South Asia where about 80% of the deaths are concentrated and the reduction in mortality is lower than elsewhere. Sub-Saharan Africa has a mortality reduction rate of 2.4% per annum which is insufficient to achieve the desired two-thirds reduction in mortality from 1990 to 2015 [2]. The main causes of mortality in these regions include neonatal conditions, malaria, pneumonia, and diarrhoea, with malnutrition being an important contributing factor. These conditions could be addressed with low cost interventions but children do not access the effective interventions promptly [1]. Many more lives could be saved through interventions that improve access to effective prevention and treatment for common childhood illnesses. In order to improve access, community-based treatment of illnesses has been recommended [3].

The home management of malaria (HMM) was one such strategy designed to provide anti-malarials for treatment of fevers at the community level. It provided prompt treatment for malaria [4] but children with non-malarial febrile illnesses like pneumonia which present with symptoms similar to malaria [5] could have been inappropriately treated with anti-malarials [6]. The World Health Organization (WHO) and UNICEF have now recommended the provision of integrated management of common childhood illnesses at the community level [7]. Following this recommendation, Uganda adopted the integrated community case management of childhood illnesses (ICCM) under which community health workers (CHWs) will provide malaria, pneumonia, and diarrhoea treatment for children less than five years [8]. It is hoped that the ICCM strategy will build on the achievements of the HMM intervention by also improving access to treatment for pneumonia and diarrhoea as well as improving treatment outcomes.

Preliminary studies on integrated malaria and pneumonia treatment in Ghana, Zambia and Uganda have assessed effects on mortality [9], treatment-seeking, drug use [10,11], timely treatment of pneumonia, and treatment outcomes where malaria diagnosis is done with the aid of rapid diagnostic tests (RDTs) [12]. However, there is still need for more evidence to inform the implementation of the ICCM strategy, including more data on child health outcomes [13,14] in various contexts. Uganda has a complex public-private mix of health care provided by government, private-not-for-profit (PNFP), and private-for-profit (PFP) facilities including private clinics, drug shops and a few hospitals. In the study setting, health care was provided by several drug shops and private clinics in addition to the CHWs, government and non-governmental organization (NGO) health units. Although private health facilities are frequently used in Uganda, they are often manned by unqualified personnel [15] and may provide poor quality of health care for childhood illnesses [16]. It was, therefore, not clear whether integrated malaria and pneumonia care from CHWs would increase prompt and appropriate treatment for pneumonia symptoms given the complex public-private mix of health care. The primary aim of this study was to determine the effect of integrated malaria and pneumonia treatment on receiving prompt and appropriate antibiotics for pneumonia symptoms. In addition, factors associated with receiving prompt and appropriate antibiotics for pneumonia symptoms were determined and treatment outcomes in the intervention and control areas were compared.

## Methods

### Study design

A follow-up study was nested within a cluster-randomized trial that compared community-based management of malaria alone to the integrated community management of malaria and non-severe pneumonia. A cluster design was considered appropriate because of the behavioural nature of the intervention. Therefore groups of villages (parishes) formed the clusters that were randomized to the intervention or control rather than individuals. Although the current study is not typically a randomized controlled trial, but was nested in an ongoing cluster-randomized trial, the consort checklist [17] has been used to guide the report as much as possible.

### Study area

The study was conducted in eastern Uganda in an area covered by the Iganga-Mayuge Health and Demographic Surveillance Site (HDSS). Iganga-Mayuge HDSS is predominantly rural with a few peri-urban areas mainly in the trading centres and Iganga Municipality. It has a total population of about 70,000 people and about 11,000 of these are children under five years. The main occupation of the people is subsistence farming. Malaria is endemic in the area with transmission peaks occurring in the rainy seasons in the months of March and September; and respiratory tract illnesses are common. The HDSS is served by one government hospital, nine public health centres, three non-governmental organization hospitals and 122 drug shops and private clinics. There were also 131 CHWs (locally known as community medicine distributors (CMDs)) who had been providing health care since 2009 under the cluster randomized trial on which the current study is based.

### The intervention

The cluster-randomized trial involved treatment of children aged 4–59 months by CHWs. CHWs have provided treatment for malaria in Uganda since 2002 under the home-based management of fever strategy. They initially used a combination of chloroquine and sulphadoxine-pyrimethamine until the malaria policy change in 2004 that led to use of artemisinin-based combination therapy (ACT) as first-line treatment for malaria [18]. The implementation of the policy was slow and use of ACT by CHWs had not yet been widely rolled out when the ICCM strategy was adopted in 2010. Therefore, there were no CHWs in the study area treating children with ACT at the time of commencement of the cluster-randomized trial in 2009. The CHWs were selected as part of the cluster-randomized trial and trained either in the management of malaria alone (which constituted the control arm) or integrated management of malaria and pneumonia (which constituted the intervention arm). The selection of CHWs has been described elsewhere [19].

### Randomization

Randomization was done by a statistician that was independent of the study using stratified block randomization. Iganga-Mayuge HDSS has 65 villages which make up 26 parishes that were divided into eight urban and 18 rural clusters (parishes). The clusters from the rural area were further grouped into three strata based on the population size of children less than five years: i) 190–320, ii) 321–390, and iii) 391 and above, resulting in six clusters in each of these strata. The clusters from the urban area were grouped into two strata based on population sizes of iv) 280–430, and v) 431 and above. Random numbers were generated in blocks of six for the rural clusters and in blocks of four for the urban clusters. From each of the three strata in the rural area three clusters were randomized to the intervention arm and three to the control arm. In each of the two strata in the urban area, two clusters were randomized to the intervention arm and two clusters to the control arm. Health facilities in the setting are equally distributed, therefore, geography and distance to formal health care was not used in the randomization process.

### Training

All CHWs received three days’ training in the management of malaria. This training was done before randomization. After randomization, the CHWs in the intervention areas received a further three days’ training in the integrated management of malaria and pneumonia. In addition, the CHWs in both arms received monthly refresher training. The details of the CHW training have been described elsewhere [19]. Prior to the training of CHWs, the district health team members were trained first in the community management of malaria and then in the integrated community management of malaria and pneumonia. This training was conducted by Ministry of Health officials together with the study investigators. Health workers in the public, non-governmental organization and private facilities also received a two-day training in integrated community management of malaria and pneumonia. They were oriented on the algorithms that were to be used by the CHWs. In addition, they were trained on investigating and documenting adverse events, and supervision and training of CHWs.

### Patient management and follow-up

The CHWs in the control arm treated children with anti-malarials, but referred children with danger signs or those with pneumonia symptoms, regardless of severity, to nearby health facilities. The CHWs in the intervention arm treated children with anti-malarials and/or antibiotics as appropriate and referred children with danger signs to nearby health facilities. No pre-referral medicines were given to children that were referred with danger signs. The diagnoses of malaria and pneumonia were made using symptoms according to the Integrated Management of Childhood Illness (IMCI) criteria [20]. The details of the treatment algorithm have been described elsewhere [11,19] but briefly, a diagnosis of “malaria” was made if the child had fever or the caregiver reported history of fever in the previous 24 hours. The CHWs did not use rapid diagnostic tests (RDTs) because they commenced their roles under the cluster-randomized trial in 2009 before WHO recommended parasite-based diagnosis for malaria in 2010 [21]. “Pneumonia” was diagnosed following the presence of cough or difficult breathing and fast breathing (≥50 breaths per minute in children aged four to 12 months and ≥40 breaths per minute in children 12 to 59 months). Breathing rates were counted using wrist watches with minutes and seconds hands. Children were classified as having severe disease if they presented with any of the four general danger signs: convulsions, repeated vomiting, lethargy/unconsciousness or failure to feed, or other danger signs: chest in-drawings, noisy breathing, severe dehydration or pallor. The CHWs were required to follow up the children they treated and refer those that did not get well to nearby health facilities. Although diarrhoea is one of the illnesses targeted by the ICCM strategy, the CHWs in this cluster-randomized trial did not treat it. This was because the trial commenced before the adoption of the ICCM strategy and was informed mainly by studies that had shown necessity to integrate malaria and pneumonia treatment due to symptom overlap. The study reported in this paper was conducted over a period of five weeks that started in mid-October to November 2011, two years after CHWs commenced their treatment roles under the cluster randomized trial.

### Study supplies

CHWs in the intervention arm were supplied with artemether-lumefantrine (AL) and amoxicillin while CHWs in the control arm were supplied with AL only. The drugs were refilled at the monthly meetings of CHWs with the cluster-randomized trial staff and formal health workers. The formulation of AL used was pre-packaged dispersible Coartem® (Novartis Pharma AG). This was supplied in packs of six tablets (20 mg artemether, 120 mg lumefantrine) for children aged 4–35 months or 12 tablets for children aged ≥ 36 months. Dispersible amoxicillin tablets (125 mg) (Medophar, India or IDA Foundation, Netherlands) were pre-packaged into three age-based doses: 6 tablets for children aged 4–11 months (one tablet twice daily for three days), 12 tablets for children 12–35 months (two tablets twice daily for three days), and 18 tablets for children 36–59 months (three tablets twice daily for three days). The dosages used in the cluster-randomized trial were chosen after some studies showed that three-day dosing was equally as effective as five-day dosing [22,23]. The amoxicillin tablets were procured in bulk and pre-packaged by a local pharmaceutical industry (Kampala Pharmaceutical Industries) into the age specific doses. The drugs were procured from the manufacturers through local pharmaceutical distributors and distributed through the district system. The CHWs in the intervention arm were supplied with watches that they used for respiratory rate counting. Neither the CHWs in the intervention arm nor the ones in the control arm had thermometers.

### Supervision

CHWs in both arms received monthly support supervision from health workers based at the nearest health facility. Details of the supervision are reported elsewhere [19].

### Study participants

All children aged 4–59 months in the intervention and control areas that were treated by CHWs for any illness were eligible for inclusion, and they were consecutively enrolled into the study. The children were identified from the CHWs’ registers and traced to their homes on the day they sought care from the CHW (day 1) or the next day for those that sought treatment late in the evening or at night (this was still within the first twenty four hours of seeking care). All enrolled children were included in the analysis for the treatment outcomes while only children with pneumonia symptoms were included in the analyses for prompt and appropriate antibiotics for pneumonia symptoms.

### Sample size

The sample size was estimated using the formula for sample size for comparing two proportions with adjustments for clustering [24]. The assumptions used were: 90% power, 5% level of significance that was two-sided, coefficient of variation of the proportions between clusters within each group of 0.4, proportions of children receiving prompt treatment for pneumonia of 13% in the control arm and 68% in the intervention arm from a previous study [12], 20% of children having pneumonia symptoms, a minimum sample size of 12 children per cluster and a drop-out rate of 10%. The coefficient of variation was assumed from literature suggesting that values of coefficients of variation are usually ≤0.25 and seldom exceed 0.5 [24], therefore 0.4 was chosen to fall within this range of acceptable values. Based on these assumptions about 111 children with pneumonia symptoms were needed in each arm, translating to about 610 children treated by CHWs in each arm.

### Definition of pneumonia symptoms

Pneumonia symptoms were defined as caregiver reports of cough accompanied by fast and/or difficult breathing similar to the definition used in MICS and DHS surveys [25]. This definition of pneumonia symptoms based on caregiver reports of symptoms was used instead of the more specific IMCI definition of pneumonia that the CHWs use, because the CHWs in the control arm did not assess and classify pneumonia symptoms and therefore had no record of which children had pneumonia symptoms. In addition, the children could not be classified by the field team of the current study because they were identified after they had received treatment from the CHWs and the symptoms and signs were likely to have changed by the time they were enrolled into the study. The definition has been used elsewhere to define presumed pneumonia [26].

### Study outcomes

The primary outcome of the study was the proportion of children receiving prompt and appropriate antibiotics for pneumonia symptoms. Prompt treatment was defined as receiving the first dose of treatment on the day of presentation of symptoms or the next day. If the child received an appropriate antibiotic for pneumonia promptly then it was classified as prompt and appropriate. The following medicines were considered appropriate for pneumonia as recommended in the national or CHWs’ treatment guidelines or the British National Formulary which is widely used in Uganda: amoxicillin, erythromycin, azithromycin, ampicillin, ampicillin plus cloxacillin, gentamicin, benzyl penicillin which could be switched to oral amoxicillin, procaine penicillin fortified (PPF), which could be switched to amoxicillin, chloramphenicol, cotrimoxazole, cefuroxime, and amoxycillin plus clavulanate [27-29]. The assessment of appropriate antibiotics did not take into account appropriate doses, frequency, and duration mainly because most of the children that received antibiotics from sources other than the CHWs would have just started these treatments by the day four evaluation. It would therefore not be possible to determine if they took the medicines appropriately and for the correct length of time. Promptness of treatment was assessed based on data collected on day one while appropriateness of antibiotics was based on day four data.

The secondary outcome was treatment outcome which included: having temperature ≥ 37.5°C or high respiratory rate on day four; received anti-malarials from other health providers after CHW-treatment or antibiotics additional to those provided by the CHWs in the intervention arm or additional to those provided by other health providers to whom children with pneumonia symptoms in the control arm were referred; hospitalization; or death [12]. Treatment outcomes were assessed on day four. Children that had any of the treatment outcomes were classified as having treatment failure. In addition, self-reported treatment failure, defined as caregiver report of non-resolution of illness symptoms, was assessed on day four.

### Data collection

The data were collected by a team of 30 experienced data collectors that were independent of the cluster-randomized trial. They were trained on the current study for one week. They were supervised by two medical officers and the first author (JNK). The data were collected using a pre-tested questionnaire (divided into two parts) that was presented in both English and Lusoga (the main local language spoken in the area) allowing the caregiver to choose between the two languages. Part one of the questionnaires was administered on day one since receiving treatment from the CHW and collected data on: socio-demographic characteristics of the caregivers and children, children’s presenting symptoms, time between onset of symptoms and seeking care, and treatment received from the CHW. Part two of the questionnaire was administered on day four to collect data on: medicines obtained from other sources, counselling reported by the caregiver, adherence to medicines, hospitalization during current illness episode, and resolution of symptoms as reported by the caregiver. Temperature and breathing rates of the children were measured on day one and day four. Axillary temperatures were measured using digital thermometers. The breathing rates were counted by the field assistants with the aid of wrist watches.

### Data management and analysis

The data were double entered into FoxPro and exported to STATA 10 (STATA Corp, College Station, TX, USA) for analysis. The baseline characteristics of the children and caregivers were summarized using descriptive statistics. Proportions of the study outcomes (prompt and appropriate antibiotics and treatment outcomes) were compared in the intervention and control arms and crude relative risks were estimated using Mantel-Haenszel methods that considered the strata used in the randomization. In order to simultaneously account for the effect of the intervention and covariates on the primary outcome, individual level analysis was done using generalized estimating equations based on Poisson regression with robust variance estimation and an exchangeable correlation matrix that accounted for clustering [30]. The individual level analysis was done after establishing that the results obtained using individual level analysis did not differ much from those obtained using cluster level analysis since there were few clusters in each arm (less than the 15 recommended for appropriate use of individual level analysis). Analysis was done on intention-to-treat basis. In order to select covariates for inclusion into the multivariate model, univariate analysis was done to determine associations between the various factors on which data were collected and receiving prompt and appropriate antibiotics using generalized estimating equations with Poisson regression. The factors with p-values less than 0.2 at univariate analysis were considered for inclusion into the multivariate model. These factors were assessed for statistical interaction and confounding with the intervention. The differences in proportions of children with fast breathing between day one and four were compared in the intervention and control arms using z-tests for comparison of proportions.

### Ethical considerations

The study was approved by Makerere University School of Public Health Higher Degrees Research and Ethics Committee (reference IRB00005876) and the Uganda National Council of Science and Technology (reference HS 898). Permission to conduct the study in the area was also obtained from the administration of Iganga-Mayuge Health and Demographic Surveillance Site and the local administration of the villages where the children were enrolled from. The caregivers of the children were informed about the study and then written informed consent was obtained from them to have their children enrolled into the study. The caregivers were given one of the two copies of informed consent documents that were signed by them and the person conducting the informed consent. Access to the data was restricted to the investigators.

## Results

**Figure 1:**
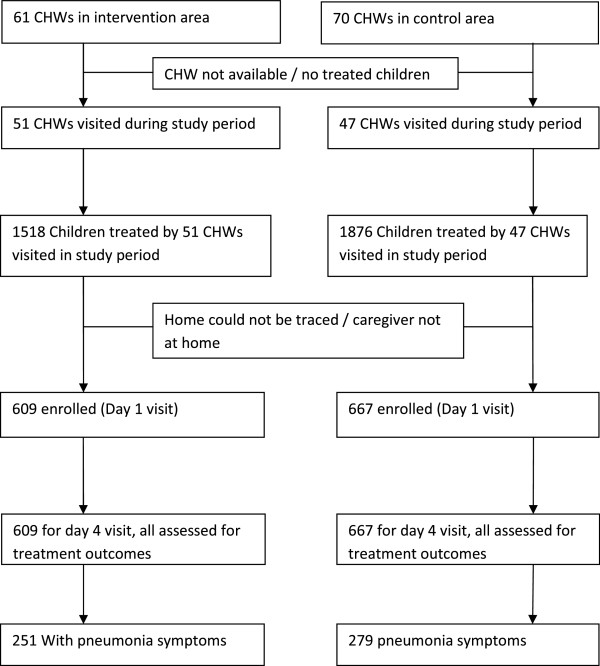
Study profile.

A total of 1,276 children were enrolled into the study (667 from the control arm, 609 from the intervention arm) as shown in the study profile (Figure 1). The children were enrolled from all clusters of the cluster-randomized trial but in varying numbers (17–138 children per cluster) depending on the number of children that were treated by the CHWs during the study period. Children were sampled from 98 of the 131 CHWs (75%) in the area.

### Demographic characteristics of the children and caregivers

The demographic characteristics of the children and caregivers were comparable in the intervention and control arms (Table 1). About half of the children in both arms were female with a mean age of about 30 months. The mean age of the caregivers was about 31 years in both arms and most of them were female, married, with primary level education. Slightly higher proportions of caregivers in the control arm were living in rural areas (90% control, 82% intervention).

**Table 1:** Demographic characteristics of children treated by CHWs and their caregivers in Iganga-Mayuge demographic surveillance site

**Characteristic**	**Intervention arm**	**Control arm**
	**N = 609**	**N = 667**
**Children’s characteristics**		
Female sex, n (%)	329 (54.0)	336 (50.4)
Mean age, months (SD)	30.4 (15.9)	31.5 (16.4)
**Caregivers’ characteristics**		
Female sex, n (%)	562 (92.3)	625 (93.7)
Age in years, mean (SD)	30.9 (9.4)	30.5 (8.7)
Education level, n (%)*		
None	59 (9.7)	62 (10.4)
Primary	381 (62.7)	450 (67.7)
Post primary	168 (27.6)	146 (22.0)
Religion, n (%)*#		
Catholic	30 (4.9)	65 (9.8)
Protestant	244 (40.1)	223 (33.5)
Muslim	310 (51.0)	335 (50.4)
Other	24 (4.0)	42 (6.3)
Marital status, n (%)**		
Married	559 (92.1)	592 (88.8)
Single	23 (3.8)	27 (4.1)
Divorced	19 (3.1)	37 (5.6)
Widowed	6 (1.0)	11 (1.7)
Rural residence, n (%)	499 (81.9)	600 (90.0)

* missing data (2 control, 1 intervention).

** missing data (2 intervention).

# significantly different.

### Illness characteristics of the children

Almost all children in both arms (97%) presented with a history of fever. However, only 15% in the intervention and 14% in the control arm had temperature ≥ 37.5°C at the time of day one data collection. Slightly more than 80% of the children in both arms had cough and about 29% in both arms reported difficult breathing. Fast breathing in children was reported by 30% of the caregivers in both arms. However, based on the respiratory rates counted by the field assistants, a higher proportion of children in the intervention arm (20%) had fast breathing on the day one visit compared to the control arm (11%). The proportions of children with reported diarrhoea, convulsions and vomiting were comparable in the two arms (Table 2).

**Table 2:** Illness characteristics of 1276 children treated by CHWs in Iganga-Mayuge HDSS

**Characteristic**	**Intervention**	**Control**	**RR (95% CI)**
History of fever, n (%)	589 (96.7)	647 (97.0)	1.00 (0.98-1.02)
Temperature ≥ 37.5^o^ C, n (%)	90 (14.8)	94 (14.1)	0.98 (0.71-1.34)
Cough, n (%)	500 (82.1)	541 (81.1)	1.00 (0.94-1.06)
Fast breathing by history, n (%)	182 (29.9)	199 (29.8)	0.99 (0.84-1.18)
Fast breathing based on respiratory rate counted by field assistant, n (%)	122 (20.0)	76 (11.4)	1.44 (1.09-1.91)
Difficult breathing by history, n (%)	174 (28.6)	194 (29.1)	0.94 (0.79-1.13)
Diarrhoea by history, n (%)	193 (31.7)	218 (32.7)	0.90 (0.76-1.07)
Convulsions by history, n (%)	40 (6.6)	40 (6.0)	0.95 (0.62-1.45)
Vomiting by history, n (%)	232 (38.2)	262 (39.5)	0.89 (0.76-1.04)

### Prompt and appropriate treatment for pneumonia symptoms

In the intervention arm, 63% of the children with pneumonia symptoms received antibiotics while in the control arm 51% were referred to health facilities. However, 45% of children with pneumonia symptoms in the intervention arm and 11% in the control arm received prompt and appropriate antibiotics (RR = 3.47, 95%CI = 2.41-4.99) (Table 3).

**Table 3:** Prompt and appropriate antibiotics among children with pneumonia symptoms

**Outcome indicator**	**Intervention**	**Control**	**RR (95% CI)**
	**n (%)**	**n (%)**	
CHW gave antibiotic/referred*	158 (63.0)	142 (50.9)	1.69 (1.23-2.32)
CHW gave antibiotic/referred**	205 (81.7)	142 (50.9)	2.01 (1.49-2.71)
Appropriate antibiotics from any source	166 (66.1)	69 (24.7)	2.41 (1.91-3.04)
Prompt and appropriate antibiotics	114(45.4)	30 (10.8)	3.47 (2.41-4.99)

* CHWs in intervention arm gave antibiotics and those in control arm referred to health facility.

** CHWs in intervention arm gave antibiotics or referred and those in control arm referred to health facility.

The association between the intervention (integrated malaria and pneumonia management) and receiving prompt and appropriate antibiotics was significant also at adjusted analysis (RR = 3.51, 95% CI = 1.75-7.03). The adjusted analysis included factors that were statistically significant or were retained as confounders, and these included: caregiver having post-primary education (RR = 1.56, 95% CI = 1.04-2.36), child presenting with no diarrhoea (RR = 1.33, 95% CI = 1.01-1.76) and living in urban residence (RR = 1.56, 95% CI = 1.20-2.00) (Table 4).

**Table 4:** Factors associated with prompt and appropriate treatment among children with self-reported pneumonia symptoms in Iganga-Mayuge DSS

**Characteristic(N)+**	**Prompt & appropriate, n(%)**	**Unadjusted RR (95%CI)**	**P-value**	Adjusted RR* (95%CI)	**P-value**
Cluster arm†					
Intervention (251)	114(45.4)	3.60 (1.92-6.73)	<0.001	3.51 (1.75-7.03)	<0.001
Control (279)	30(10.8)	1.00		1.00	
Primary caregiver					
Male (35)	15(42.9)	1.45(0.85-2.50)	0.18		
Female (495)	129(26.1)	1.00			
Education, caregiver†					
≤Primary (415)	96 (23.1)	1.00	0.001	1.00	0.03
Post primary (114)	48 (42.1)	1.73 (1.25-2.40)		1.56 (1.04-2.36)	
No occupation/housewife/farmer (434)	97(22.4)	1.00	0.013		
Other (96)	47(49.0)	1.85 (1.14-3.01)			
Child had diarrhoea†					
Yes (193)	44(22.8)	1.00	0.006	1.00	0.04
No (337)	100(29.7)	1.46 (1.12-1.90)		1.33 (1.01-1.76)	
Child had convulsions					
Yes (43)	10(23.3)	1.00	0.09		
No (486)	133(27.4)	1.31 (0.96-1.78)			
Child was vomiting					
Yes (219)	69(31.5)	1.20	0.11		
No (310)	75(24.2)	1.00 (0.96-1.52)			
Residence†					
Urban (79)	43(54.4)	2.08 (1.25-3.45)	0.005	1.56 (1.20-2.00)	0.001
Rural (451)	101(22.4)	1.00		1.00	

+N represents the number in each category.

* Adjusted analysis includes all variables with †.

### Treatment outcomes

There was no difference in overall treatment failure among children in the intervention and control arms (RR = 0.88, CI = 0.67-1.18). However, children in the control arm were more likely to have temperatures above 37.5°C on day four compared to children in the intervention arm (4% versus 1%). In addition, there was a higher difference between proportions of children with fast breathing on day one and four in the intervention (9.2%) compared to control areas (4.2%, p = 0.01). There were no deaths reported. Perceived treatment failure was significantly lower in the intervention arm compared to the control arm (RR = 0.58, 95%CI = 0.46-0.73) (Table 5).

**Table 5:** Treatment outcomes of children treated by CHWs in Iganga-Mayuge DSS

**Characteristic**	**Intervention**	**Control**	**RR (95% CI)**
*All children treated by CHWs*	N = 609	N = 667	
Overall treatment failure	90 (14.8%)	101 (15.1%)	0.88 (0.67-1.18)
Received additional* antibiotics	7 (1.2%)	13 (2.0%)	0.77 (0.32-1.86)
Received additional** anti-malarials	4 (0.7%)	10 (1.5%)	0.44 (0.15-1.28)
Hospitalization	10 (1.6%)	9 (1.4%)	1.47 (0.64-3.38)
Temperature ≥ 37.5°C on day 4	7 (1.2%)	23 (3.5%)	0.29 (0.11-0.78)
Had fast breathing on day 4	66 (10.8%)	48 (7.2%)	1.29 (0.87-1.92)
Difference in fast breathing, day 1& 4†	9.2%	4.2%	p-value = 0.01
Self reported treatment failure	83 (13.7%)	170 (25.5%)	0.58 (0.46-0.74)
*Children with malaria symptoms*			
Characteristic	Intervention	Control	RR (95% CI)
	n = 589	n = 647	
Overall treatment failure in malaria	84 (14.3%)	99 (15.3%)	0.85 (0.64-1.13)
Received additional* antibiotics	6 (1.0%)	13 (2.0%)	0.65 (0.26-1.65)
Received additional** anti-malarials	3 (0.5%)	10 (1.6%)	0.35 (0.11-1.11)
Hospitalization	9 (1.5%)	9 (1.4%)	1.36 (0.59-3.16)
Temperature ≥ 37.5°C on day 4	7 (1.2%)	22 (3.4%)	0.30 (0.11-0.81)
Had fast breathing on day 4	62 (10.5%)	48 (7.4%)	1.22 (0.82-1.82)
Difference in fast breathing, day 1& 4,†	9.5%	4.0%	p-value = 0.006
Self reported treatment failure	78 (13.3%)	168 (26.0%)	0.56 (0.44-0.71)
*Children with self reported pneumonia symptoms*			
Characteristic	Intervention	Control	RR (95% CI)
	n = 251	n = 279	
Overall treatment failure in “pneumonia”	43 (17.1%)	56 (20.1%)	0.77 (0.52-1.13)
Received additional* antibiotics	2 (0.8%)	10 (3.6%)	0.30 (0.08-1.21)
Received additional** anti-malarials	0 (0)	8 (2.9%)	-
Hospitalization	4 (1.6%)	3 (1.1%)	2.42 (0.60-9.84)
Temperature ≥ 37.5°C on day 4	5 (2.0%)	11 (3.9%)	0.41 (0.10-1.75)
Had fast breathing on day 4	34 (13.6%)	25 (9.0%)	1.30 (0.76-2.21)
Difference in fast breathing, day 1& 4 †	18.3%	3.6%	p-value < 0.001
Self reported treatment failure	22 (8.8)	74 (26.5)	0.35 (0.22-0.56)

* Additional antibiotics received after CHW treatment in intervention arm or after treatment from other source where children were referred in control arm. The CHWs in the control arm did not have antibiotics and children with pneumonia symptoms are expected to receive antibiotics from other health providers.

** Additional anti-malarials received after treatment by CHWs.

† difference between percentage of children with fast breathing on day one and four, p-value comparing difference in intervention and control.

Among children with pneumonia symptoms, children in the control arm were more likely to receive additional anti-malarials (3% control, 0% intervention).

### Additional medicines received by children

The use of additional medicines was infrequent. Among the children that received additional medicines after receiving care from the CHW, paracetamol was the most frequently used medicine in children from both the intervention (76%) and control (74%) arms followed by cotrimoxazole (14% intervention, 16% control) and cough syrups (10% intervention, 6% control) (Table 6).

**Table 6:** Additional medicines received by children after treatment by CHW

**Medicine**	**Intervention**	**Control**
	**(n = 42)**	**(n = 96)**
Paracetamol	32 (76.2)	71 (74.0)
Cotrimoxazole*	6 (14.3)	15 (15.6)
Cough syrups	4 (9.5)	6 (6.3)
Amoxicillin*	1 (2.4)	5 (5.2)
Chlorphenramine	6 (14.3)	5 (5.2)
Metronidazole	2 (4.8)	4 (4.2)
Quinine†	0 (0)	3 (3.0)
Ampicillin*	0 (0)	2 (2.0)
Artemether-lumefantrine†	3 (7.1)	2 (2.0)
Benzylpenicillin*	0 (0)	2 (2.0)
Chloramphenicol*	0 (0)	2 (2.0)
Chloroquine†	1 (2.4)	2 (2.0)
Combination flue medicines	0 (0)	2 (2.0)
Dexamethasone	1 (2.4)	2 (2.0)
Unknown antimalarial syrup†	0 (0)	2 (2.0)
Ampiclox*	0 (0)	1 (1.0)
Anti-emetic	1 (2.4)	1 (1.0)
Cetrizine	0 (0)	1 (1.0)
Erythromycin*	0 (0)	1 (1.0)
Ibuprofen	1 (2.4)	1 (1.0)
Mebendazole	0 (0)	1 (1.0)
ORS	1 (2.4)	1 (1.0)
Vitamins	1 (2.4)	0 (0)

* considered as additional antibiotics.

† considered as additional anti-malarials.

## Discussion

This study has demonstrated that integrated community case management of malaria and pneumonia increases prompt and appropriate treatment for self-reported pneumonia symptoms. In addition, children in the control arm were more likely to have a high temperature on day four of treatment seeking. There was a greater reduction in fast breathing in children in the intervention compared to the control arm.

This study has demonstrated that integrated community case management of malaria and pneumonia increases prompt and appropriate antibiotics for self-reported pneumonia symptoms in a rural area with a large number of private clinics and drug shops but which may be manned by unqualified health providers. These findings are similar to those reported in a study in Zambia where prompt and appropriate treatment was higher among children treated by CHWs that could treat both malaria and pneumonia [12]. This similarity is found despite the differences in study designs and contexts of the Zambian and the current study. In the Zambian study, RDTs were employed for malaria diagnosis which was likely to have improved the accuracy of the illness classification. The Zambian study was also in more controlled conditions where children were recruited as they sought care from the CHWs compared to the current study which is better classified as an effectiveness study because children were recruited after they sought care and were therefore not aware of their participation in the study at the time of seeking care. In addition, the current study was done in a setting with several drug shops and clinics which are likely to be additional sources of antibiotics. As expected in such a setting, the difference between the intervention and control arms was lower compared to that in the Zambian study though not significantly.

The proportion of children that received prompt and appropriate antibiotics was higher among children whose caregivers had received post primary education. This may be due to better recognition of symptoms among the more educated caregivers or better affordability of treatment resulting in more prompt care seeking. The study in Zambia found a tendency to more prompt and appropriate treatment among caregivers with primary or secondary education compared to those that did not have any education although it was not statistically significant [12]. Differences in receiving prompt and appropriate antibiotics may point to inequalities that may exist in accessing health care in persons of different social standing. Low education has been associated with low income and low knowledge on aspects that affect use of health services [31].

The children in urban areas were more likely to receive prompt and appropriate antibiotics. This may be due to easier access to treatment in the urban areas. It may also be related to caregivers’ knowledge of disease symptoms. There were higher proportions of caregivers with post primary education in the urban areas and these may have better health knowledge than the caregivers with primary or no education [32] that were mostly living in the rural areas. Caregivers in urban areas have better knowledge of childhood illness and also have better health-seeking behaviour [33]. Disparities in access to health care have been observed between rural and urban areas [34].

There was no difference in overall treatment failure among all children treated by CHWs in the intervention and control areas, similar to a study in Zambia [12]. However, contrary to the Zambian study where the proportion of children with persistent fever, fast or difficult breathing at follow up did not differ, the current study found a higher proportion of febrile children in the control arm on day four despite both groups having similar proportions of children with high temperature on day one. In addition, although the children in the intervention arm had a higher proportion of children with fast breathing on day one and day four (although not significant on day four), there was a greater reduction in the proportions of children with fast breathing in the intervention arm. The differences seen in the two studies could have been a result of the way the outcomes were presented. In the Zambian study persistent fever and fast or difficult breathing were grouped together which could have masked outcome-specific differences. The initial higher fast breathing in the intervention arm could have resulted from more caregivers in the control arm whose children had pneumonia symptoms bypassing the CHWs and taking their children directly to other health providers. Similar differences in proportions of children with respiratory symptoms were also noted in the study in Zambia. These findings suggest improved treatment outcomes when children can access effective treatments promptly. Many of the children in the control arm that got antibiotics received cotrimoxazole against which high levels of resistance of pneumonia-causing bacteria have been reported [35,36]. This finding supports the need to have the effective medicines for common childhood illnesses at the first level of care because the children need them. This may prevent the delays experienced in receiving necessary treatment.

There was no difference in overall treatment failure among children with pneumonia symptoms contrary to the Zambian study [12] where children classified as having pneumonia in the intervention arm were less likely to have treatment failure compared to their counterparts in the control arm. The difference in findings could be a result of how pneumonia was classified in the two studies. The current study used caregiver reports of pneumonia symptoms whereas in the Zambian study the classification of pneumonia was based on the CHWs classification; which may have led to lower misclassification of children as having pneumonia when they did not [37]. This would make it easier to detect differences in treatment failure in children treated by CHWs who treated and those that did not treat pneumonia. However, reduction in fast breathing (based on respiratory rates measured by the data collectors) between day one and four among children with pneumonia symptoms in the intervention arm of the current study (18%) compared to reduction in fast breathing in the control arm (3.6%) is even higher than that seen among all children treated by CHWs (9.2% versus 4.2% respectively).

While this study assessed only symptoms and treatment of malaria and pneumonia, current WHO recommendations are to integrate also diarrhoea treatment into integrated community case management. The findings from this study support this recommendation to incorporate ORS and zinc since a considerable proportion of children had diarrhoea (about 30%) but only two children received ORS and likely no child zinc.

### Methodological issues

Self-reported symptoms were used to classify children into whether they had possible pneumonia and thus children may have been misclassified. The IMCI guidelines, which CHWs use to make classifications, define pneumonia as cough or difficult breathing and fast breathing [20]. The classification of fast breathing is based on age-specific thresholds of breath counts which could not be ascertained based on the caregiver reports. It was not possible to take the baseline assessments of respiratory rates in the children because they were located on day one of treatment-seeking and for some of the children the illness may have changed by the time they were seen. In addition, the CHWs’ records could not be used for classification of pneumonia because in the control arm, the CHWs do not assess and classify pneumonia symptoms. They had no record of which children had presented with pneumonia symptoms. Use of caregivers’ reports to classify pneumonia symptoms has been used by Multiple Indicator Cluster Surveys (MICS) and Demographic and Health surveys (DHS) as well as other studies to identify children with possible pneumonia in the community [25,26], but it is likely to over-estimate the proportion of children with true pneumonia. Therefore, the results of appropriate treatment obtained may reflect over-treatment with antibiotics [37]. The desirable treatment rate may therefore not be 100%, until a more specific indicator has been found [37]. However, this measure though unspecific, does not introduce differential bias between the study arms, thus allowing for relative comparisons. A further classification of pneumonia was done based on the respiratory rate assessments of children on day one by the field assistants. The results showed an even bigger difference in the proportion of children that received prompt and appropriate treatment for pneumonia symptoms between the intervention and control arms compared to the results obtained using self reported pneumonia symptoms. This suggests that the use of self reported pneumonia symptoms in the current study could have led to under- rather than over-estimation of the effect of integrated malaria and pneumonia management because self-reported pneumonia symptoms may be more sensitive but less specific than the respiratory rate assessed by data collectors on day one.

The analysis presented was done at the individual level whereas it would have been better to do cluster level analysis since there were fewer than 15 clusters in each arm. However with analysis at the cluster level it would have been difficult to simultaneously control for other variables [30]. The results obtained from analysis at the cluster and individual levels at unadjusted analysis were compared and found similar. Adjusted analysis based on residuals for the primary outcome was done and found that it was still significant. The analysis done at the individual level with adjustments for the cluster randomization to show effects of other variables has, therefore, been presented. In addition, there were imbalances in the number of children treated in the clusters which could have resulted in loss of power. However, the effect may have been minimal since a difference in the main outcome variable has been detected between the intervention and control areas. Nevertheless, other associations may have been undetectable.

There were no buffer zones between the intervention and control areas which may have led to dilution of the intervention effects since some caregivers in the control areas could have accessed antibiotics from the intervention areas. However, a three-fold difference between the intervention and control areas is still demonstrated on receiving prompt and appropriate antibiotics.

Treatment failure may have been underestimated because danger signs of illness were not assessed on day four. This could have underestimated treatment effects in the integrated care arm.

Another limitation of this study is that it did not include all children treated by the CHWs in the cluster-randomized trial but only assessed those that were treated over a period of five weeks. There may have been variations in outcomes of children treated over the whole year.

## Conclusion

Implementation of integrated community case management is an effective approach in improving access to prompt and appropriate antibiotics for pneumonia symptoms and may improve treatment outcomes. This may reduce mortality in children less than five years since pneumonia is one of the major killers of children globally.

## Abbreviations

ACT: Artemisinin-based combination therapy; AL: Artemether-lumefantrine; CHWs: Community health workers; CMDs: Community medicine distributors; HDSS: Health and demographic surveillance site; HMM: Home management of malaria; ICCM: Integrated Community Case Management of Childhood Illnesses; IMCI: Integrated Management of Childhood Illness; NGO: Non Governmental Organization; ORS: Oral rehydration salts; PFP: Private for Profit; PNFP: Private not for Profit; PPF: Procaine Penicillin Fortified; RDTs: Rapid diagnostic tests; RR: Relative risk; SD: Standard deviation; UNICEF: United Nations Children’s Fund; WHO: World Health Organization.

## Competing interests

The authors declare that they have no competing interests.

## Authors’ contributions

JNK, TA, SP, CK, and ER took part in: designing the study, development of study tools, data analysis and manuscript writing. KM took part in development of study tools. JNK and KM participated in data collection. All authors read and approved the final manuscript.
